# Focal-adhesion kinase regulates the sialylation of *N*-glycans *via* the PI4KIIα-PI4P pathway

**DOI:** 10.1016/j.jbc.2023.105051

**Published:** 2023-07-13

**Authors:** Yuhan Sun, Tomoya Isaji, Yoshiyuki Oyama, Xing Xu, Jianwei Liu, Hisatoshi Hanamatsu, Ikuko Yokota, Nobuaki Miura, Jun-ichi Furukawa, Tomohiko Fukuda, Jianguo Gu

**Affiliations:** 1 Division of Regulatory Glycobiology, Institute of Molecular Biomembrane and Glycobiology, Tohoku Medical and Pharmaceutical University, Sendai, Miyagi, Japan; 2 Department of Orthopedic Surgery, Hokkaido University Graduate School of Medicine, Sapporo, Japan; 3 Division of Glyco-Systems Biology, Institute for Glyco-Core Research, Tokai National Higher Education and Research System, Nagoya, Japan; 4 Division of Bioinformatics, Niigata University Graduate School of Medical and Dental Sciences, Niigata, Japan

**Keywords:** focal adhesion kinase, *N*-glycosylation, integrin, regulation, sialic acid

## Abstract

Sialylation is a terminal glycosylated modification of glycoproteins that regulates critical biological events such as cell adhesion and immune response. Our previous study showed that integrin α3β1 plays a crucial role in regulating the sialylation of *N*-glycans. However, the underlying mechanism for the regulation remains unclear. This study investigated how sialylation is affected by focal adhesion kinase (FAK), which is a critical downstream signal molecule of integrin β1. We established a stable FAK knockout (KO) cell line using the CRISPR/Cas9 system in HeLa cells. The results obtained from lectin blot, flow cytometric analysis, and MS showed that the sialylation levels were significantly decreased in the KO cells compared with that in wild-type (WT) cells. Moreover, phosphatidylinositol 4-phosphate (PI4P) expression levels were also reduced in the KO cells due to a decrease in the stability of phosphatidylinositol 4-kinase-IIα (PI4KIIα). Notably, the decreased levels of sialylation, PI4P, and the complex formation between GOLPH3 and ST3GAL4 or ST6GAL1, which are the main sialyltransferases for modification of *N*-glycans, were significantly restored by the re-expression of FAK. Furthermore, the decreased sialylation and phosphorylation of Akt and cell migration caused by FAK deficiency all were restored by overexpressing PI4KIIα, which suggests that PI4KIIα is one of the downstream molecules of FAK. These findings indicate that FAK regulates sialylation *via* the PI4P synthesis pathway and a novel mechanism is suggested for the integrin-FAK-PI4KIIα-GOLPH3-ST axis modulation of sialylation in *N*-glycans.

Focal adhesion kinase (FAK) is known to function as a 125-kDa tyrosine kinase that participates in the phosphorylation of different targets and is also known as a platform in the formation of protein complexes (1). FAK contains a four-point-one, ezrin, radixin, and moesin (FERM) domain at the N-terminal domain, a central kinase domain, and a C-terminal focal adhesion targeting (FAT) domain. The FERM domain has three lobed subdomains to assist with autophosphorylation (2) and binding to other substrates and phosphatidylinositol 4,5-bisphosphate (3). The FAT domain is determined by its subcellular localization connection to other adhesion plaque components such as talin (4) and paxillin (5) that attach to the tails of integrin β1. The Y397 site is the autophosphorylated site of FAK, which interacts with the SH2 domain of several proteins such as Src (6) and phosphatidylinositol 3-kinase (PI3K) (7) to activate the MAPK and Akt signaling pathways. FAK is known to overexpress in numerous cancers (8) such as lung cancer (9), osteosarcoma (10), and ovarian cancer (11). FAK is a significant mediator of integrin-mediated signal transduction that connects the intracellular and extracellular regulation of biological functions such as cell adhesion, migration, and invasion (12).

Glycosylation is one of the most common post-translational modifications in eukaryotic proteins. According to estimates, at least 50% of proteins are glycosylated (13), and *N*-glycans participate in various biological activities. Integrins composed of α and β subunits are highly modified by *N*-glycans (14). Bisected *N*-glycans on integrin modified by *N*-acetylglucosaminyltransferase III (GnT-III) inhibit cell migration and metastasis (15). By contrast, the *N*-glycans on integrin modified by α1,6-fucosyltransferase (Fut8) (16), *N*-acetylglucosaminyltransferase V (GnT-V) (17), and sialyltransferases (STs) (18, 19, 20) enhance tumor malignancy. Sialylation is one of the essential modifications, and sialic acid covalently bonds to the terminal end of glycans (21). There are 20 Golgi-localized STs that catalyze three types of sialylation: α2,3 or α2,6 bonding to galactose (Gal) or to *N*-acetylgalactosamine (GalNAc), and α2,8 bonding to another sialic acid. Although overlapping functions usually occur, sialylation nonetheless plays a critical role in regulating cell- and microenvironment-specific processes (22). The expression of sialylation is dynamic, and changes usually relate to immune functions (23). Sialic acid as the receptor on glycoproteins can be recognized by virus hemagglutinin and neuraminidase, which are essential to viral endocytosis and its propagation (24). Siglecs, which are the family of sialic acid-binding lectins, display specificity for distinct sialylated glycan structures (22). Increased α2,6-sialylation mediates immunosuppression by binding to the Siglecs on immune cells (25). Moreover, the changes in sialylation are usually related to cancer development (26). Hypersialylation has been identified in colon cancer cells, hepatocarcinoma cells, and breast cancer cells (27). In particular, upregulation of sialylation on β1-integrin usually occurs in cancer cells and affects cell growth and proliferation (20), cell migration (18), and cell invasion (19). In our previous studies, we found that the oncogenic protein Golgi phosphoprotein 3 (GOLPH3) regulates sialylation, and this activity is related to its interaction with sialyltransferases such as ST6Gal1 or ST3Gal4 and phosphatidylinositol 4-phosphate (PI4P) (28). Moreover, the complex formations of phosphatidylinositol 4-kinase-IIα (PI4KIIα) and integrin α3β1 play essential roles in the upregulation of sialylated *N*-glycans, and the sialylation levels of *N*-glycans are significantly suppressed by either α3 knockout (KO) or PI4KIIα knockdown (29). However, the underlying mechanism for the regulation by integrin remains elusive.

In this study, we established FAK knockout (KO)- and FAK-restored cell lines to investigate the molecular mechanism between integrin and sialylation. We found that FAK up-regulates PI4P expression by enhancing the stability of PI4KIIα, which increases sialylation. These findings further support a novel concept for the regulation of sialylation such that sialylation levels are controlled by both transcriptional glycogenes and by the integrin-FAK-PI4KIIα-GOLPH3-ST axis.

## Results

### A deficiency of FAK downregulates sialylation levels

**Figure 1:**
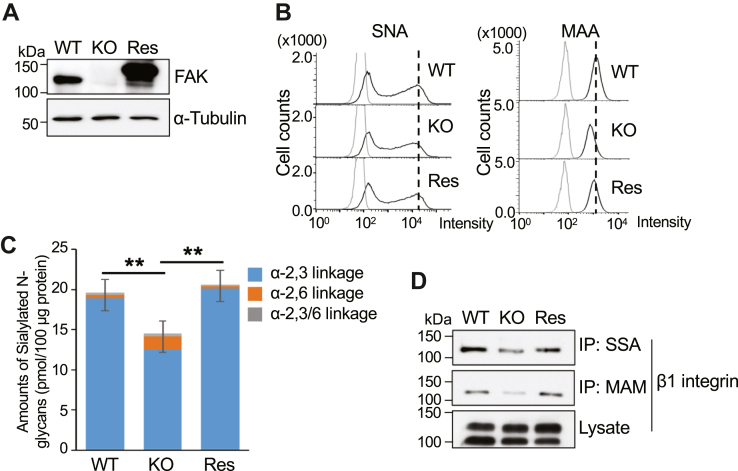
**Effects of FAK KO on the sialylation of *N*-glycans.***A*, cell lysates were extracted from the WT, FAK KO, and restored using FAK genes in the FAK-KO (Res) HeLa cells. Equal amounts of cell lysates were loaded into 7.5% SDS-PAGE gel to detect the expression of FAK. *B*, comparison of the sialylation levels on the cell surface among the WT, FAK KO, and Res HeLa cells *via* flow cytometry analysis. The same numbers of cells were incubated with SNA and MAA, which preferentially recognized α2,6- or α2,3-sialylation, respectively. *C*, comparison of the expression levels of sialylated *N*-glycans that belong to three different categories (*N*-glycans with α2-3 linked sialic acids only, α2-6 linked sialic acids only, and the mixture of both linkages) in 100 μg proteins of cell lysate among the three cell types with MALDI-TOF MS. The data were obtained from four independent samples. Comparisons of the FAK KO cells for each group following total sialylation showed significant differences, which was performed using one-way ANOVA with Tukey’s *post hoc* analysis as the mean ± SD. ∗∗*p* < 0.01. *D*, equal amounts of proteins were immunoprecipitated (IP) with SSA-agarose or MAM-agarose and detected by anti-integrin β1 antibody. The expression levels of endogenous β1 in the cell lysates were also western blotted with anti-β1 antibody.

As described above, our previous study showed that integrin α3β1 is essential for the sialylation of *N*-glycans. FAK, a significant downstream mediator of integrin β1, is vital in the integrin-mediated signal pathway. Here, we used the CRISPR/Cas9 system to first establish the FAK KO HeLa cell lines and selected two clones that were checked *via* Western blot (Fig. S1*A*, upper panel) and confirmed by genomic sequence analysis (Fig. S1*A*, lower panel). The sialylation levels were detected *via* flow cytometric analysis using either *Sambucus nigra agglutinin* lectin (SNA/SSA) or *Maackia amurensis* lectin (MAA/MAM), as these preferentially recognize α2,6-sialylation and α2,3-sialylation, respectively. Both sialylations were downregulated in FAK KO cells compared with the wild-type (WT) HeLa cells. The degree of α2,3-sialylation decrease was more significant than that of α2,6-sialylation (Fig. S1*B*). To understand whether this observation is universal, we used the CRISPR/Cas9 system to establish 293T FAK KO cell lines (Fig. S1*C*, upper panel), which were confirmed by genomic sequence analysis (Fig. S1*C*, lower panel). Both sialylation levels were consistently decreased in the KO cells compared with that in the WT cells detected by flow cytometric analysis (Fig. S1*D*). The degree of α2,3-sialylation decrease was smaller than that of α2,6-sialylation in 293T cells. These results suggest that FAK KO downregulates sialylation. We primarily used the HeLa KO-2 clone in the subsequent experiments involving the establishment of stable cells by viral transduction and used 293T KO-2 clone in complementary experiments in which transient transfection was involved. We first tested whether the overexpression of FAK in the HeLa KO cells can rescue the sialylation. The expression levels of FAK were examined by Western blotting with an anti-FAK antibody (Fig. 1*A*), and the sialylation levels were detected by flow cytometric analysis using SNA and MAA lectin. The sialylation level could be recovered in the Res cells (Fig. 1*B*).

In addition, we used MALDI-TOF MS spectra to verify the sialylation levels of *N*-glycans, and 85 *N*-glycans were detected (GlycoPOST) and divided into seven types (Table 1). The total amounts of *N*-glycans were not altered among the three cell types. Still, the parts of sialylated *N*-glycans were significantly lower in the KO cells than that in either WT or Res cells (Fig. 1*C*). When focusing on the linkages, curiously, the amounts of α2,3-sialylation in the KO cells were lower, while the expression levels of α2,6-sialylation seemed to be higher than those in either the WT or Res cells (Fig. 1*C*), which may suggest competition between α2,3-sialylation and α2,6-sialylation for modification. Furthermore, the sialylation levels of signal protein were examined. Integrin β1 is known to be a highly *N*-glycosylated membrane protein (30, 31, 32, 33). Both α2,3- and α2,6-sialylation levels were decreased in the KO cells as evidenced by the immunoprecipitation of SSA-agarose and MAM-agarose (Fig. 1*D*). These results clearly show that the total sialylation levels are suppressed by FAK KO, which can be rescued by restoration of the FAK gene and indicates the importance of FAK for sialylation regulation.

**Table 1:** Expression levels of *N*-glycans in HeLa cells analyzed by MALDI-TOF MS spectra

*N*-glycan class (pmol/100 μg protein ± SD)	WT	FAK KO	Res
Pauci-mannose	3.3363 ± 0.3616	5.0862 ± 0.8510	4.0287 ± 0.7827
High-mannose	98.7420 ± 7.2006	120.3126 ± 13.7415	110.2682 ± 9.6342
Neutral complex glycan	8.3421 ± 0.6851	4.4005 ± 0.6696	7.6751 ± 0.7625
Acid complex glycan	17.6258 ± 0.4262	13.7104 ± 1.4353	18.6302 ± 1.7614
Neutral hybrid glycan	2.4108 ± 0.2602	1.0924 ± 0.1434	1.4637 ± 0.1495
Acid hybrid glycan	2.0802 ± 0.6347	1.1195 ± 0.0940	1.3630 ± 0.2021
Others	1.0319 ± 0.1080	0.5159 ± 0.0437	0.5839 ± 0.1208
Total	129.4671 ± 9.2844	143.5096 ± 15.6266	140.6022 ±10.8217

**Figure 2:**
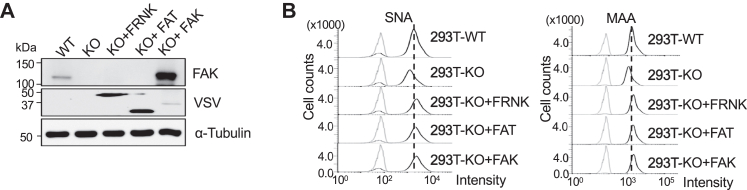
**Effects of FAK variants on sialylation of *N*-glycans in 293T cells.***A*, the FAK-KO 293T cells were transfected with or without VSV-FRNK, VSV-FAT, or FAK. Post-transfection 48 h, the expression levels of FAK were detected with anti-FAK or anti-VSV antibodies. α-Tubulin was used as a loading control. *B*, comparison of the sialylation levels on the cell surface by flow cytometry analysis among the WT, FAK KO, and the KO cells overexpressed with VSV-FRNK, VSV-FAT, or FAK plasmid as described above. The same numbers of cells were collected and stained with SNA and MAA lectin.

To establish whether the kinase activity of FAK is essential in the sialylation of *N*-glycans, we transfected the FAK-related non-kinase (FRNK) domain and FAT domain into the FAK KO 293T cells, which was confirmed by western blotting with indicated antibodies (Fig. 2*A*). The sialylation levels were determined *via* flow cytometric analysis with SNA and MAA (Fig. 2*B*). Surprisingly, both α2,6- and α2,3-sialylation levels could be restored by not only WT FAK but also by FRNK and FAT mutants. These results suggest that the FAK kinase domain is not essential for sialylation regulation but that the complex formation between integrin and FAK is vital.

### The levels of PI4P and complex formation between GOLPH3 and sialyltransferases were decreased in FAK KO cells

**Figure 3:**
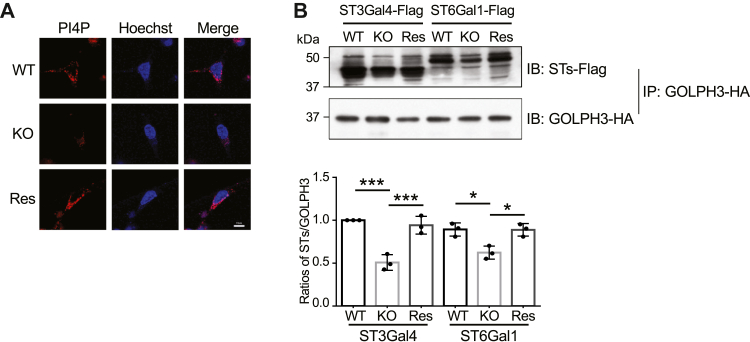
**Effects of FAK on PI4P expression and complex formation between GOLPH3 and sialyltransferases.***A*, comparison of expression and localization of PI4P detected by mRFP-FAPP1 among the indicated HeLa cells. Scale bars, 10 μm. *B*, effects of FAK on complex formation between GOLPH3 and STs. Equal amounts of proteins of three types of HeLa cells (WT, FAK KO, and Res) co-transfected with GOLPH3-HA and ST3Gal4-Flag or ST6Gal1-Flag, were immunoprecipitated with anti-HA-agarose, and the immunoprecipitates were detected using anti-Flag antibody. The experiments were independently repeated three times. The relative intensities were calculated by the intensities of the total sialyltransferases (ST3Gal4 or ST6Gal1)-Flag against GOLPH3-HA. The ratio of the intensities of the ST3Gal4-Flag against GOLPH3-HA was set as 1.0. All values reflect one-way ANOVA with Tukey’s *post hoc* analysis as the mean ± SD. ∗*p* < 0.05; ∗∗∗*p* < 0.001.

GOLPH3 has been identified as an oncogenic protein in cancer localized to the Golgi (34), and in a previous study, we found that its interactions with PI4P and sialyltransferases (ST3Gal4 or ST6Gal1) are essential for the regulation of sialylation (28). PI4P can be recognized by the four-phosphate adaptor protein 1 (FAPP1), which was fused to a red fluorescent protein (mRFP) as a monitor for the expression and distribution of PI4P (35). The expression levels of PI4P were decreased in KO cells compared with that in WT cells, while these were significantly restored in the Res cells (Fig. 3*A*). PI4P plays an essential role in regulating sialylation possibly through interactions between GOLPH3 and sialyltransferases, since GOLPH3 contains a pleckstrin homology (PH) domain, which binds to PI4P (36). Therefore, we verified the complex formation between GOLPH3 and ST3Gal4 or ST6Gal1, which are two major sialyltransferases for α2,3- and α2,6-sialylation of *N*-glycans, respectively. The results showed that the association between GOLPH3 and ST3Gal4 or ST6Gal1 was significantly decreased in the HeLa KO cells and could be restored in the Res cells (Fig. 3*B*). These phenomena also appeared in 293T cells. However, the association between ST6Gal1 and GOLPH3 showed a stronger interaction in 293T cells (Fig. S2*C*), which could explain the more significant decrease in α2,6-sialylation in 293T cells compared with α2,3-sialylation (Fig. S1*D*). These data suggest that FAK regulates the complex formation between GOLPH3 and ST3Gal4 or ST6Gal1 through PI4P.

### The expression levels of PI4KIIα were suppressed in FAK KO cells

**Figure 4:**
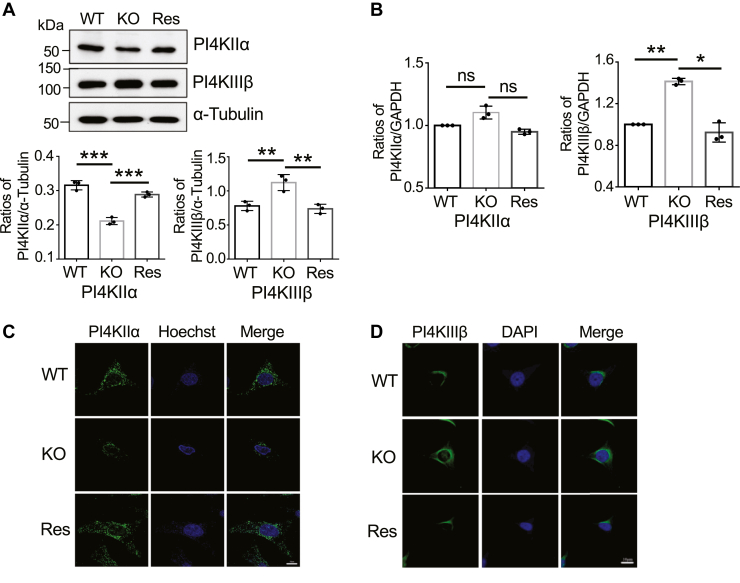
**Effects of FAK on expression levels of PI4KIIα and PI4KIIIβ in HeLa cells.***A*, expression levels of PI4KIIα and PI4KIIIβ were detected *via* Western blot. Equal proteins were subjected to 7.5% SDS-PAGE gel and verified by indicated antibodies. α-Tubulin was used as a loading control. Data were quantified using Image J software and obtained from three independent experiments. All values reflect one-way ANOVA with Tukey’s *post hoc* analysis as the mean ± SD. ∗∗*p* < 0.01; ∗∗∗*p* < 0.001. *B*, the mRNA levels of PI4KIIα and PI4KIIIβ in the WT, FAK KO, and Res HeLa cells were detected by qPCR. GAPDH was used as an internal control. All values were normalized to that of the GAPDH. The ratio of the intensities of WT was set as 1.0. Data represent the mean ± SD from three independent experiments. *p* > 0.05; ∗*p* < 0.05; ∗∗*p* < 0.01. *C*, the expression vector containing GFP-PI4KIIα was transfected into the WT, FAK KO, or Res HeLa cells. After transfection for 72 h, the fluorescence for PI4KIIα expression was detected using a ZEISS LSM 900 confocal microscope. Scale bars, 10 μm. *D*, the expression levels of endogenous PI4KIIIβ were detected by immunostaining with an anti-PI4KIIIβ antibody. Scale bars, 10 μm. ns, no significance.

PI4P is catalyzed by phosphatidylinositol 4-kinase (PI4K) *via* the phosphorylation of substrate PI phospholipids. Different PI4Ks have different localizations and exhibit different functions in mammalian species (37). PI4KIIα and phosphatidylinositol 4-kinase-IIIβ (PI4KIIIβ) are known to contribute primarily to Golgi-localized PI4P (34), and PI4KIIα plays a significant role in forming the cellular PI4P in the post-trans-Golgi network (38). However, PI4KIIIβ primarily localizes to the Golgi, nucleus, and ER, and PI4KIIα has been detected in the plasma membrane, endosomes, and TGN (37). To explore the mechanism of the decreased expression of PI4P in FAK KO cells, we detected the expression levels of PI4KIIα and PI4KIIIβ. The results obtained from western blotting with indicated antibodies showed that the expression levels of PI4KIIα were significantly decreased in the KO cells, while the expression levels of PI4KIIIβ were increased (Figs. 4*A* and S2*A*). These changes could be canceled in the Res cells. Then we verified the mRNA levels of PI4KIIα and PI4KIIIβ. Quantitative room-temperature PCR (RT-PCR) analysis showed that the mRNA levels of PI4KIIα had no significant differences among WT, KO, and Res cells, while the expression levels of PI4KIIIβ were increased in the KO cells compared with that in the WT and Res cells (Figs. 4*B* and S2*B*).

To further confirm the location and expression levels of PI4KIIα and PI4KIIIβ, we overexpressed the PI4KIIα tagged with GFP in the three types of cells and found that the fluorescent intensities of PI4KIIα were decreased in the KO cells compared with that in the WT and Res cells (Fig. 4*C*). Conversely, the expression levels of endogenous PI4KIIIβ immunostaining with anti-PI4KIIIβ antibody were increased in the KO cells compared with that in the WT or Res cells (Fig. 4*D*), which support the results obtained from Western blot analysis (Fig. 4*A*). These results suggest that the decrease in PI4P could be caused by a decrease in the expression levels of PI4KIIα rather than that of PI4KIIIβ in the KO cells.

### The stability of PI4KIIα was attenuated in FAK KO cells

**Figure 5:**
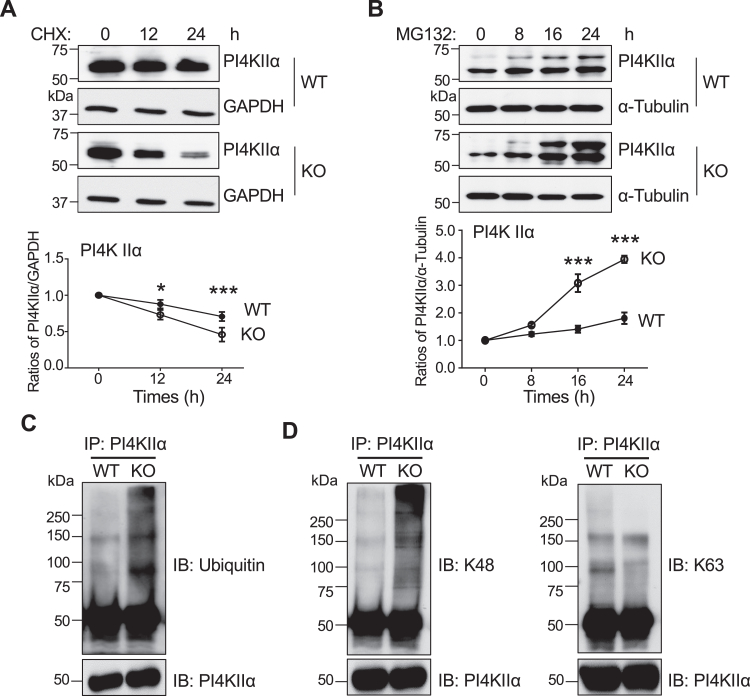
**Effects of FAK on PI4KIIα stability.***A*, the WT and FAK KO HeLa cells were treated with 50 mM cycloheximide (CHX), a protein synthesis inhibitor. *B*, 30 μM MG132, a proteasome inhibitor, for indicated times. Equal cell lysates were used for western blotting with an anti-PI4KIIα antibody. GAPDH or α-Tubulin was used as a loading control. The relative values were calculated by the density of PI4KIIα at each indicated time point against the density of that at time 0 h (without treatment). The ratio at the 0 h point was set as 1.0. Values represent the mean ± SD from three independent experiments. ∗*p* < 0.05; ∗∗∗*p* < 0.001. Equal amounts of proteins of WT and FAK KO HeLa cell lysates were immunoprecipitated with anti-PI4KIIα antibody. The immunoprecipitates were western blotted with anti-ubiquitin antibody (*C*), and anti-K48 or anti-K63 ubiquitin antibody (*D*). The endogenous PI4KIIα was detected by the anti-PI4KIIα antibody as a loading control.

To further investigate the underlying mechanism for the decreased expression of PI4KIIα in the HeLa KO cells, we used cycloheximide (CHX) to examine the stability of PI4KIIα, a protein synthesis inhibitor in eukaryotic cells (39). The decay of PI4KIIα in KO cells was significantly faster than that in WT cells in both HeLa cells (Fig. 5*A*) and 293T cells (Fig. S3*A*). Conversely, the stability of PI4KIIα was further examined using MG132, a proteasome inhibitor, which reduces protein degradation when conjugated to ubiquitin (40). The endogenous expression levels of PI4KIIα were gradually increased during the treatment with MG132 in the WT cells. The accumulation of PI4KIIα was significantly increased in the KO cells compared with that in the WT cells in both HeLa cells (Fig. 5*B*) and 293T cells (Fig. S3*B*).

Ubiquitination is one of the essential modifications related to protein degradation. K48 and K63 are different lysine residues that regulate various biological functions in ubiquitin molecules (41). K48 linkage is the most prevalent of ubiquitin chain types. It enhances degradation by targeting proteins for proteasomal degradation, whereas the K63 linkage is related to endocytosis, DNA damage responses, and immune responses as a regulatory signal to protect target proteins (42). To further confirm the enhanced ubiquitination of PI4KIIα in the HeLa KO cells, we immunoprecipitated the endogenous PI4KIIα in HeLa WT and FAK KO cells and verified the ubiquitination levels of K48 and K63. The ubiquitination of PI4KIIα was increased in the KO cells compared with that in WT (Fig. 5*C*) and 293T cells (Fig. S3*C*). The K48-linked ubiquitination of PI4KIIα seemed to be at a higher level in the KO cells compared with that in WT cells. By contrast, the K63-linked ubiquitination of PI4KIIα was decreased in the KO cells compared with that in WT cells (Figs. 5*D* and S3*D*). Moreover, after treatment with MG132 in 293T cells, the phenomenon became more obvious (Fig. S3*D*). Taken together, these results demonstrate that a deficiency in FAK reduces the stability of PI4KIIα through an enhancement of the proteasomal degradation pathway.

### Over-expressed PI4KIIα reversed the decrease in sialylation caused by a deficiency in FAK

**Figure 6:**
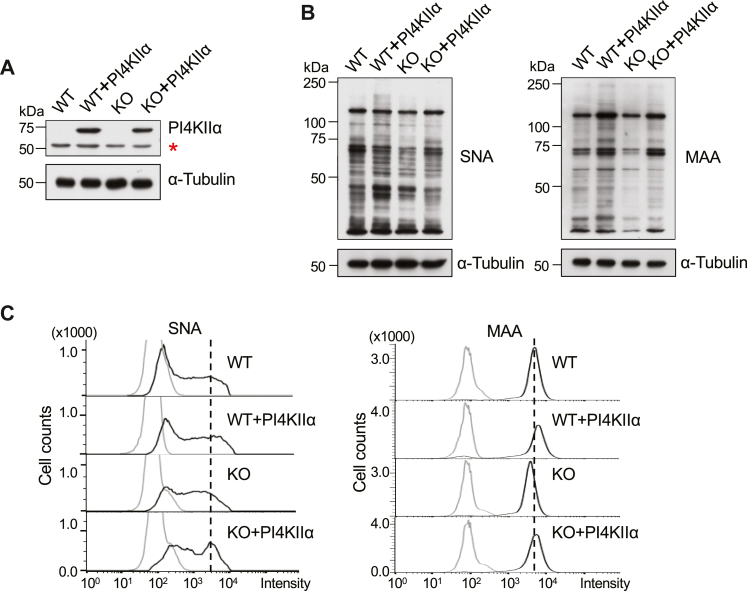
**Overexpression of PI4KIIα restored the sialylation levels that were suppressed in FAK KO cells.***A*, WT and FAK KO HeLa cell lines that over-expressed PI4KIIα were established and verified by western blotting with an anti-PI4KIIα antibody. The asterisk indicates endogenous PI4KIIα bands. α-Tubulin was used as a loading control. *B*, the sialylation levels were examined by lectin blotting with SNA and MAA. Equal proteins were subjected to 7.5% SDS-PAGE gel and blotted with SNA and MAA lectins. α-Tubulin was used as a loading control. *C*, the sialylation levels on the cell surface were detected by flow cytometry analysis using SNA and MAA lectins.

To confirm whether the decrease in sialylation was related to the down-regulation of PI4KIIα in the HeLa FAK KO cells, we established a stable overexpression of PI4KIIα in HeLa, WT, and FAK KO cells and confirmed expression *via* Western blot (Fig. 6*A*). The sialylation levels were examined by lectin blot (Fig. 6*B*) and flow cytometric analysis (Fig. 6*C*) with SNA or MAA. Both α2,6- and α2,3-sialylation were increased in the over-expressed PI4KIIα cells compared with the corresponding WT or KO cells, which further suggests that PI4KIIα is a downstream factor of FAK in regulating sialylation. Likewise, these results proved that PI4KIIα could reverse the decrease in sialylation caused *via* FAK deficiency.

### Overexpression of PI4KIIα enhanced cell migration that was suppressed in FAK KO cells

**Figure 7:**
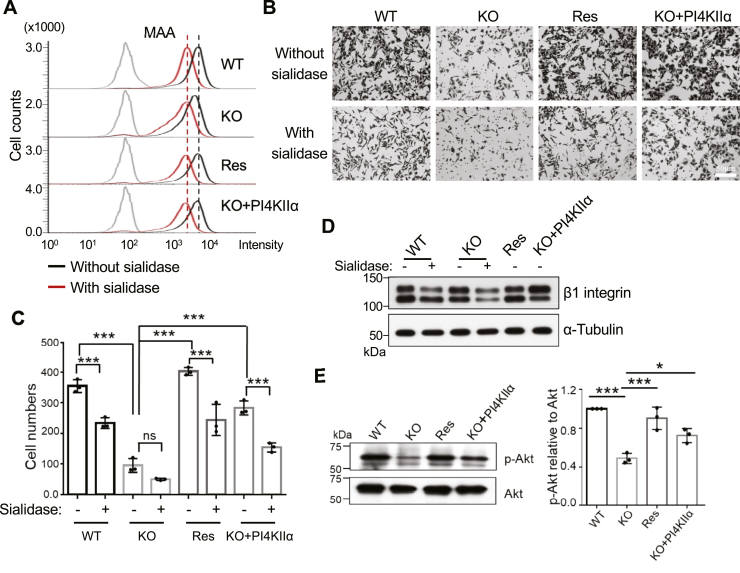
**Comparison of cell migration and cellular signaling among the four different HeLa cells.** Equal numbers of the four cell lines were treated with or without sialidase as described in “Experimental procedures”. *A*, after treatment with sialidase, cells were collected, and the sialylation levels on the cell surface were detected by flow cytometry analysis using MAA lectin. The *red* and *black lines* indicated that the cells were treated with or without sialidase, respectively. The *gray line* represented autofluorescence (no staining) as each negative control. *B*, cell migration was examined by the transwell assay described in “Experimental procedures”. There are representative fields for each indicated cell after migration in the transwell assay. *C*, quantitative analysis of cell migration. The data were obtained by counting migrated cells from three random fields. All values represent the mean ± SD. *p* > 0.05; ∗∗∗*p* < 0.001. *D*, equal proteins with or without sialidase were loaded and blotted by Western blot using anti-β1 integrin antibody. α-Tubulin was used as a loading control. *E*, the phosphorylated Akt and total Akt expression levels among the four cells were detected by western blotting with the indicated antibodies. Data were quantified using Image J software and obtained from three independent experiments. The ratio of the intensities of WT was set as 1.0. All values reflect one-way ANOVA with Tukey’s *post hoc* analysis as the mean ± SD. ∗*p* < 0.05; ∗∗∗*p* < 0.001. ns, no significance.

FAK participates in the integrin-mediated formation of adhesion plaque, which regulates cell adhesion and migration. FAK KO suppresses the cell migration of fibroblasts (43). In addition, the previous excellent study clearly shows that sialylation of β1 integrin plays an important role in cell migration (44). We wondered whether the altered surface sialylation drove cell migration in the FAK (Res) and PI4KIIα HeLa cells. We treated the cells using sialidases, which cleave all kinds of sialylation on cell membranes and then conducted detection *via* flow cytometric analysis using MAA lectin (Fig. 7*A*). A transwell migration assay was used to detect the cell-migration abilities. Cell migration was significantly suppressed in WT, FAK (Res), and PI4KIIα cells after treatment with sialidases. However, the effects of sialidase treatment on FAK KO seemed small (Fig. 7, *B* and *C*, lower panel), which could have been due to the significant impact of FAK KO on cell migration. Moreover, the results of Western blot showed that the molecular weight of β1 integrin was slightly lower in FAK KO cells than in either WT or KO+PI4KIIα cells, which is similar to the case of β1 in the WT cells treated with sialidase (Fig. 7*D*). Unexpectedly, cell migration was also enhanced in HeLa KO cells in which FAK or PI4KIIα was overexpressed, even when these cells were treated with sialidase. (Fig. 7, *B* and *C*). This result means that both FAK and PI4KIIα could enhance cell migration and that the sialylation that is regulated by them partially enhances migration. The PI3K-Akt signal pathway plays a vital role in integrin-mediated cell migration and cancer invasion. The levels of phosphorylated Akt were significantly reduced in the HeLa KO cells compared with that in the HeLa WT cells (Fig. 7*E*). The decreased levels of phosphorylated Akt in KO cells were reversed by overexpression not only of FAK but also of PI4KIIα (Fig. 7*E*). These results show that PI4KIIα is a downstream molecule of FAK that can partially and significantly reverse cell biological functions impaired by a deficiency in FAK.

## Discussion

**Figure 8:**
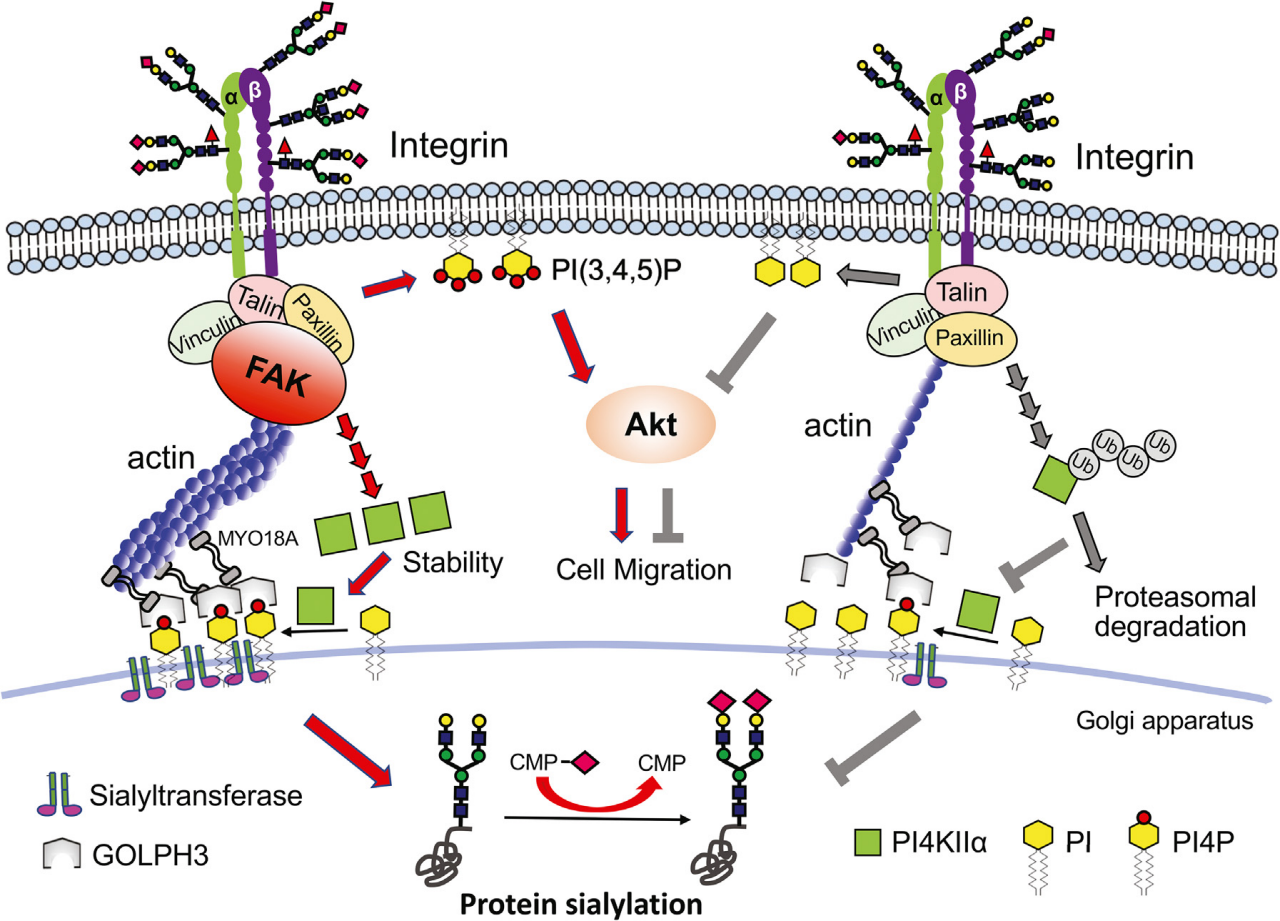
**Schematic diagram of the regulatory mechanism for sialylation by the integrin-FAK-PI4K IIα-GOLPH3-ST axis.** It is well known that FAK is a critical downstream molecule of integrin β1 (45). GOLPH3 is known to bind with myo18, which interacts with actin filaments through its motor domain (36). This study showed that the integrin-FAK complex could significantly contribute to the stability of PI4KIIα, which enhanced PI4P production and subsequently increased the complex formation between GOLPH3 and sialyltransferases on the Golgi membrane. Finally, the intact structure might promote and increase efficiency for sialylation on *N*-glycans (*left panel*). Conversely, suppressed expression of α3β1 integrin (29) or deficiency of FAK (the present study), or knockdown of GOLPH3 (28) or PI4KIIα (29) decreased sialylation on *N*-glycans. The lack of FAK down-regulated PI4KIIα expression levels *via* the proteasomal degradation pathway leads to suppression in the complex formation among GOLPH3, PI4P, and sialyltransferases (*right panel*). The present study demonstrates that the integrin-FAK-PI4KIIα-GOLPH3-ST axis is a crucial pathway to regulate sialylation on glycoproteins, which may, in turn, influence cellular signaling and cancer metastasis.

In the present study, we first determined how FAK regulates the sialylation of *N*-glycans, which should help clarify the underlying mechanism and explain why integrin β1 regulates sialylation (29). We found the following three points: (I) the deficiency of FAK down-regulates the sialylation and PI4P levels, which is due to a decrease in PI4KIIα stability *via* the proteasomal degradation pathway; (II) FAK KO decreases the complex formation between GOLPH3 and sialyltransferases; and, (III) restoration of FAK or overexpressed PI4KIIα reverses the decreases in sialylation, PI4P expression, and cell migration caused by FAK KO. These results demonstrate how FAK regulates the alternation in sialylation through the integrin-FAK-PI4KIIα-GOLPH3-ST axis (Fig. 8).

FAK is activated by integrin clustering and is critical for assembling the complexes within focal adhesions (FAs). The regulatory mechanism of FAK function is related to its autocatalytic tyrosine kinase activity combined with the SH2 domain to phosphorylate downstream substrates (6). Another regulatory mechanism depends on the FRNK domain, a 42-kDa protein, that is related to the formation of focal adhesions with tensin and paxillin and successively forms a complex with integrin β1 (45). In the present study, the latter point could be a key mechanism involving the sialylation regulation by FAK since both FAT and FRNK are able to sufficiently reverse decreases in sialylation levels caused by FAK KO (Fig. 2). There is evidence suggesting a correlation between FAT and paxillin (46). The suppression of paxillin expression affects the Golgi morphology (47). Additionally, GOLPH3 has been found to bind with myo18 (36) and sialyltransferases (48) to affect the Golgi apparatus. These findings confirm our conclusion that FAK plays a crucial role in regulating sialylation on *N*-glycans (Fig. 8), which is controlled by the integrin–FAK complex rather than by kinase activation of FAK.

It is noteworthy that FAK could upregulate both α2,6- and α2,3-sialylation of β1 integrin (Fig. 1*D*). The effect of FAK on sialylation is similar to that of others such as PI4KIIα, integrin α3, and GOLPH3 in HeLa or MDA-MB231 cells (28, 29). It is curious, however, that mass spectrometry analysis revealed that the expression levels of α-2,6-linked sialylated *N*-glycans were higher in FAK KO cells than those in either the WT or Res cells (Fig. 1*C*). Thus far, we cannot precisely explain the underlying mechanisms, but the phenomenon suggests substrate competition among sialyltransferases. It could be explained by a downregulation of α2,3-sialylation, which is a significant sialylation for target proteins in most differentiated cells and leads to the provision of more donor substrates (CMP-sialic acid) for ST6Gal1, which would increase the levels of α2,6-sialylation (Fig. 1*C*). Substrate competition among sialyltransferases such as ST3Gal3, ST3Gal4, ST3Gal6, and ST6Gal1 has been observed in HeLa cells (49), but this is not always the case. For example, both α2,6- and α2,3-sialylation were suppressed in integrin β1 in the KO HeLa cells (Fig. 1*D*). Considering there is substrate competition among sialyltransferases, the mechanism of sialylated regulation is complicated. Our previous study showed that the α2,6-sialylation levels of integrin β1 increased in the ST3Gal4 KO cells, which is a crucial enzyme for the α2,3-sialylation of *N*-glycans (49). Therefore, we could speculate that FAK KO downregulates both forms of sialylation, but the outcome may depend on different levels of sialyltransferases and target glycoproteins. Thus, substrate competition may occur in some situations, which depends on the expression levels of each sialyltransferase, plausible regulators, and/or the target protein. Thus far, apart from sialylation we cannot explain the reasons for changes such as complex glycans being reduced and high-mannose glycans being increased in FAK-KO HeLa cells compared with that in WT cells (Table.1, Fig. S4). These data suggest that FAK could participate in such forms of regulation through different pathways, but this will require further study.

Sialylation is catalyzed through the transfer of CMP-sialic acid and modifies the terminal linkage of *N*-glycans *via* sialyltransferases. The mechanisms of altered sialylation in cancer cells have been reported. Several studies have shown that some oncogenes contribute to the regulation of sialylation. The proto-oncogene Ras controls α2,6-sialylation by impacting the transcription of ST6Gal1 (50). The transcription factor c-Myc up-regulates α2,3-sialylation by affecting the transcription of ST3Gal1/3/4 (51). However, this is not always the case. In previous studies, we found that sialylation is regulated through interactions between GOLPH3 and STs rather than by the upregulation of these sialyltransferase genes at transcriptional levels (28). Moreover, the expression of integrin α3β1, as well as that of PI4KIIα, is required for regulation (29). In the present study, FAK was a critical downstream molecule of integrin β1 and also played an essential role in regulation. The deficiency of FAK decreased sialylation through the downregulation of PI4P expression and through the interaction between GOLPH3 and ST3Gal4/ST6Gal1 (Figs. 3*B* and S2*C*). These results further establish that the complex formation of GOLPH3-PI4P-ST is essential for sialylation and also shows that the complex recruits STs to a specific domain in the Golgi membrane to increase efficiency for sialylation (Fig. 8).

It is well known that PI4P is formed at the Golgi by PI4Ks that catalyze the phosphorylation of substrate PI phospholipids (37). Among PI4Ks, PI4KIIα localized on the Golgi catalyzes about 50% of the PI4P and in concert with PI4KIIIβ plays an important role in Golgi-related trafficking (52). In this study, we again noticed that the expression levels of PI4P were mainly catalyzed by PI4KIIα rather than by PI4KIIIβ since FAK KO significantly decreased PI4P levels through downregulation of PI4KIIα protein expression (Fig. 4), which was supported by the previous observation in which PI4KIIα knockdown decreased PI4P and sialylation expression (29). The FAK KO increased the decay of PI4KIIα, which was confirmed by cycloheximide, a protein synthesis inhibitor. Curiously, the protein levels of PI4KIIα were decreased. By contrast, the PI4KIIIβ levels for both proteins and genes were increased in the FAK KO cells, following restoration by the re-expression of FAK (Fig. 4*A*). In addition, the overexpression of PI4KIIα restored both sialylation and cell migration as well as cellular signaling such as that by phospho-Akt, which was blocked in the FAK KO cells (Fig. 7). Based on these observations, we could speculate that PI4KIIα is a critical regulator for sialylation and is a downstream FAK molecule.

To understand the decreased stability of PI4KIIα in the FAK KO cells, we investigated the proteasomal degradation pathway using MG132 and found that the FAK KO greatly enhanced the degradation pathway. Ubiquitination is a common protein modification that involves covalent ligation of ubiquitin (Ub) molecules in the lysine residue on target proteins (53). The different types of Ub chains such as K6, K11, K27, K29, K33, K48, and K63 regulate various cellular processes (54). K48 chains, for example, regulate protein levels by signaling a target protein for degradation by the proteasome (54), whereas K63 chains serve nonproteolytic roles in intracellular protein trafficking, transcription, inflammation, and DNA repair (55). Here, we found upregulation of K48 polyUb and downregulation of K63 polyUb in the FAK KO cells compared with that in WT cells (Figs. 5 and S3), which may explain the decreased stability of PI4KIIα in the FAK KO cells. Of course, underlying mechanisms such as identifying which E3 ligase is responsible for the proteasomal degradation of PI4KIIα in the FAK KO cells, will require further study.

In conclusion, the results of this study establish FAK as a novel regulatory mechanism for the *N*-glycans in sialylation. These results and previous observations reinforce the notion that sialylation is regulated not only by the transcriptional levels of glycogenes but also by the integrin-FAK-PI4KIIα-GOLPH3-ST axis that is beyond transcriptional regulation, which suggests a new level of significance for highly expressed FAK and GOLPH3 in many solid cancers.

## Experimental procedures

### Antibodies and reagents

The experiments were performed using the following antibodies and reagents: Focal adhesion kinase (FAK; 610088), anti-PI4-kinase β (PI4KIIIβ; 611816), and integrin β1 (610468), which were obtained from BD Transduction Laboratories. Biotinylated *S. nigra agglutinin* (SNA; B-1305) and an ABC kit (PK-4000) were from Vector Laboratories; *M. amurensis* (MAM) lectin (#BA-s7801-2) was from EY Laboratories. The antibody against HA (1867423) was from Roche Applied Science. The antibodies against FLAG (F1804) and α-Tubulin (T6199) and the peroxidase-conjugated secondary antibody against mouse IgG (AP124P) were from Sigma. The antibodies against PI4KIIα (ab71824), GOLPH3 (ab91492), ubiquitin (linkage-specific K48) (ab140601), and ubiquitin (linkage-specific K63) (ab179434) were from Abcam. The anti-hST6GAL1 (AF5924) anti-GFP antibody (600-401-215) was from Rockland Immunochemicals, Inc. The anti-Akt (9272) and p-Akt (4060) antibodies and the peroxidase-conjugated secondary antibody against rabbit (7074S) were from Cell Signaling Technology. The anti-GAPDH antibody (sc-25778) was from Santa Cruz Biotechnology. The goat anti-mouse IgG Alexa Fluor 568 and streptavidin conjugate Alexa Fluor 647 antibody were from Invitrogen. The cycloheximide (CHX, 037-20991) was from Wako; MAM-Agarose (J310) and SSA-Agarose (J318) were from J-oil Mills; anti-HA-tag pAb-Agarose (561-8), anti-GFP (Green Fluorescent Protein), and mAb-Magnetic Beads (D153-11) were from MEDICAL & BIOLOGICAL LABORATORIES CO, LTD. The transwell (BD BioCoat control inserts, 8.0-μm inserts) was from BD Biosciences. Tris (2-carboxyethyl) phosphine hydrochloride (TCEP) was from Sigma. Peptide-*N*-Glycosidase F (PNGase F) was from Roche. TDisialyloctasaccharide (A2GN1) was from Tokyo Chemical Industry. The sialidase (P0720L) was from New England Biolabs, Inc. BlotGlyco beads were from Sumitomo Bakelite Co Ltd. The SialoCapper-ID Kit was from the Shimadzu Corporation. Aminooxy-functionalized tryptophanylarginine methyl ester (aoWR) was prepared as previously described (56). MultiScreen Solvinert 0.45 μm low-binding hydrophilic polytetrafluoroethylene plates were purchased from Merck Millipore. MassPrep HILIC μElution plates were obtained from the Waters Corporation. Other solvents and reagents were of the highest grade commercially available.

### Cell lines and cell culture

The HeLa and 293T cells (RIKEN Cell Bank, Japan) were cultured in DMEM with 10% fetal bovine serum under a standard atmosphere at 37 °C and 5% CO_2_.

### Expression vectors and transfection

The plasmid of VSV-tagged FRNK and FAT were obtained as previously described (57, 58). Briefly, FAK cDNA was polymerous chain reaction (PCR)-amplified from glioma cells, and the sequences of FRNK and FAT were PCR-amplified from HA-FAK, which was kindly provided by Dr Kenneth Yamada (NIDCR/NIH). Vesicular stomatitis virus epitope-tagged FRNK was constructed by inserting the vesicular stomatitis virus epitope at the 5′ end of the FRNK, and the full sequence was verified *via* DNA sequencing. The cDNA of HA-tagged GOLPH3 was provided by Dr Lynda Chin, Institute for Applied Cancer Science, the University of Texas MD Anderson Cancer Center (59). The cDNAs of STs were provided by Dr Hisashi Narimatsu from the National Institute of Advanced Industrial Science and Technology, Japan. The resultant cDNAs were inserted into pENTR 1A and tagged with 3× FLAGs at the C terminus using the infusion method (Takara Bio) and were confirmed *via* DNA sequencing, as previously described (29). All plasmids we used in this study were established by the Gateway cloning system from Invitrogen. The resultant plasmids were used for transfection with PEI MAX (Polysciences Inc). In brief, an equal number of cells (5 × 10^6^) were seeded 12 h before the transfection. The resultant plasmids (10 μg) were dissolved in 0.5 ml Opti-MEM (Gibco) for 5 min as buffer A. Simultaneously, PEI MAX (30 μg dissolved in 30 μl of 0.2 M HCl) was dissolved in 0.5 ml Opti-MEM at RT for 5 min as buffer B. After mixing buffers A and B at RT for 15 min, the mixture was gently transferred into a cell-cultured dish. After incubation for 6 h, the conditioned medium was replaced by a fresh standard culture medium for further incubation for 48 h.

### Establishment of FAK KO cells and restored cells with FAK or overexpressed with PI4KIIα in the FAK KO cells

The pSpCas9(BB)-2A-GFP (PX458) plasmid was acquired from Addgene (PX458: Addgene #48138). FAK-KO cells were constructed *via* guide RNA (5′- GAA TCA GTT ACC TAA CGG ACA -3′) targeted to human FAK genes localized adjacent to Cas 9 in the pSpCas9(BB)-2A-GFP vector and sorting the GFP-expressing cells. The stable HeLa-FAK-KO cell line and 293T-FAK-KO cell line were established by electroporating cells according to the manufacturer's recommendations (Amaxa cell line Nucleofector kit; Lonza, Basel, Switzerland). Post-transfection 24 h, the cells with positive fluorescence were sorted using FACSAria II (BD Bioscience), diluted, and then seeded on 96-well plates to obtain single clones. After incubation for 3-weeks, those single clones were expanded. We extracted the genome and validated the CRISPR target region *via* PCR amplification using the following primers: Forward primer, 5′- TTTCCTTCTGCCACCTCTGTTCTC -3′; Reverse primer, 5′- GCACCTAGACTTTCTCTTACTTGTCCC -3′. Sequencing was then accomplished with the reverse primer.

The stable over-expressions of FAK or PI4KIIα in the FAK KO or WT HeLa cell lines were established *via* virus infection with CSII-EF-Rfa-GFP-FAK plasmid or CSII-CMV-GFP-PI4KIIα plasmid (29). GFP-tagged FAK and PI4KIIα were obtained through standard PCR protocols. The subcloned cDNAs were transferred into CSII-EF-Rfa (28) *via* the gateway system for lentivirus production. The resultant vectors (CSII-EF-GFP-PI4KIIα or FAK) were transfected into 293T cells with packaging plasmids during the preparation of viruses. After transfection for 48 h, the conditioned media containing lentivirus was collected and clarified by filtration through a 0.45 μm filter. The supernatants obtaining the target virus were then used to infect the HeLa FAK KO cells. The infected cells were sorted twice *via* FACSAria II, which could ensure the sorted cells were overexpressed.

### Immunoprecipitation, Western blot, and lectin blot

Cells were washed three times with cold PBS and then lysed in the cell lysate buffer (20 mM Tris-HCl, pH 7.4, 150 mM NaCl, 1% Triton X-100), including protease and phosphatase inhibitors (Nacalai Tesque) for 30 min on ice. Protein concentrations of the cell lysates were determined using a BCA protein assay kit (Pierce). For immunoprecipitation, the same amounts of proteins from each cell lysate were immunoprecipitated using either anti-GFP mAb-magnetic beads or anti-HA pAb-agarose at 4 °C for 2 h with rotation. And then, these immunoprecipitates were washed twice with TBS and detected by western blotting with indicated antibodies.

Western blot and lectin blot were performed by loading equal amounts of proteins (10 μg) or immunoprecipitants into either 7.5 or 10% sodium dodecyl sulfate-polyacrylamide gel electrophoresis (SDS-PAGE) at 100 V followed by transfer to polyvinylidene difluoride membranes (PVDF, Millipore Sigma). After blocking with 5% bovine serum albumin (BSA) for lectin blot or 5% non-fat dry milk for Western blot for 1 h at RT, the membranes were incubated overnight with either the indicated primary antibodies or biotinylated lectins. After washing, the blots were incubated with the appropriate secondary antibodies. According to the manufacturer's instructions, immunoreactive bands were detected using an immobilon Western Chemiluminescent HRP Substrate (Millipore).

### Flow cytometry analysis

Cells were detached by brief exposure to 0.25% trypsin containing 1 mM EDTA and resuspended at 1 × 10^6^ cells/ml density. After washing with ice-cold PBS, equal amounts of cells were incubated with biotinylated lectins (SNA or MAA) in 0.1% BSA in PBS for 30 min on ice. Subsequently, the cells were incubated with streptavidin conjugate Alexa Fluor 647 (1:500) for 25 min at RT in the dark. Then, the cells were washed and resuspended in 1 ml MACS buffer (PBS containing 0.1% BSA). The fluorescence intensities were detected by Attune flow cytometer (BD Biosciences) and analyzed using FlowJo software.

### RNA extraction and real-time PCR for the detection of mRNA expression levels

RNAs were extracted by Trizol reagent (Invitrogen), and 1 μg of total RNAs was transcribed into cDNA using a PrimeScript RT reagent with a gDNA Eraser (Takara, Japan). The primer sequences are listed in Table 2. Real-time PCR analyses were performed using a One-Step PrimeScript RT-PCR Kit (Takara). The real-time PCR conditions were as follows: activated SYBR Ex Taq for 10 s at 95 °C, then 40 cycles of denaturation were performed for 5 s at 95 °C followed by annealing for 30 s at 60 °C.

**Table 2:** Primer sequences for RT-PCR

Gene name	Forward primer	Reverse primer
PI4KIIα	ctccagcggaagctacttcg	tccacttaggattaagatgccca
PI4KIIIβ	cccagatcagggaggaggaa	aagagccggttgccaatgta
GAPDH	cggagtcaacggatttggtcgta	agccttctccatggtggtgaagac

### *N*-glycan preparation

Cell pellets included 100 μg of proteins treated with TCEP and 2-iodoacetamide in 100 mM ammonium bicarbonate containing 0.1% Triton-X (Sigma). After reductive alkylation, the proteins were digested with trypsin at 37 °C for 16 h. The reaction mixture was heated at 90 °C for 10 min to inactivate the trypsin. Deglycosylation was carried out by the addition of 2 U of PNGase F. Released *N*-glycan analysis was subjected to a glycoblotting procedure in combination with the aminolysis-SALSA method as previously described (60, 61). In brief, released *N*-glycans (100 μg protein) containing 10 pmol A2GN1 as an internal standard were captured on BlotGlyco beads. Then, unreacted hydrazide groups on beads were capped by acetylation. The aminolysis-SALSA method was performed on beads to protect and discriminate the sialylated *N*-glycan isomers. Next, these amidated *N*-glycans were released and labeled with aoWR *via* transamination. Excess aoWR reagent was removed using a HILIC μElution plate, followed by MALDI-TOF MS analysis.

### Annotation of *N*-glycans from the MALDI-TOF MS spectra

The *N*-glycans were automatically annotated and quantified from the MALDI-TOF MS spectra *via* the use of the TAG List and the Expression program in the Toolbox Accelerating Glycomics (TAG) suite (62, 63). A heat map of the glycan expression levels was created using R version 3.6.3.

### Immunofluorescence

Equal numbers of cells were seeded into glass-bottom dishes. After washing the cells, 4% paraformaldehyde (PFA) was used to fix the cells for 30 min. Next, cells were treated with 0.1% TritonX-100 in PBS for 10 min and incubated with 5% BSA in PBS at RT for 2 h to block non-specific staining. The cells were stained with anti-PI4KIIIβ antibody (1:300 dilution) overnight at 4 °C, washed with PBS, and incubated with secondary antibody (goat anti-mouse IgG Alexa Fluor 568) for 2 h and DAPI at RT for 8 min in the dark with detection accomplished using a ZEISS LSM 900 confocal microscope.

### Cell migration (transwell assay)

According to a procedure established in a previous report (29), a transwell assay was briefly performed using 24-well plates (BD BioCoat control inserts, 8.0-μm inserts; BD Biosciences) as follows. The number of cells (5 × 10^5^ cells) was counted and incubated for 1 h at 37 °C with or without sialidase. After treatment with sialidase, 5 × 10^4^ cells were counted and seeded into 400 μl of serum-free medium that was placed into the upper chamber. The lower chamber of a 24-well plate contained 500 μl of 10% FBS culture medium. Following 16 h of incubation, cells were fixed across the pores with methanol for 15 min and stained with a crystal violet solution for 15 min.

### Cells treated with sialidase

Cells were detached by brief exposure to 0.25% trypsin containing 1 mM EDTA and washed with PBS 2 times. The cells (1 × 10^6^) were resuspended in 20 μl Glycobuffer (50 mM CaCl_2_, 500 mM sodium acetate, pH 5.5) diluted with 180 μl PBS containing 0.1% BSA, and incubated with or without 2 units sialidase (New England Biolabs, Inc) for 1 h at 37 °C with gently shaking according to the manufacturer's instructions. After incubation, the cells were washed with ice-cold PBS and then used for flow cytometry analysis and cell migration assay.

### Statistical analysis

All data are presented as the mean ± SD obtained from at least three independent experiments. Statistics were analyzed using GraphPad Prism 5.0 software (GraphPad Software, Inc). One-way ANOVA with Tukey’s *post hoc* testing along with an unpaired Student *t* test were performed for statistical analysis. A probability value of *p* was considered as follows: ns (no significance), *p* > 0.05; statistically significant, ∗*p* < 0.05; ∗∗*p* < 0.01; ∗∗∗*p* < 0.001.

## Data availability

Glycomic raw data for glycan-structure analysis by MALDI-TOF MS was deposited to the GlycoPOST; announced ID: GPST000329.

## Supporting information

This article contains supporting information.

## Conflicts of interest

The authors declare no conflicts of interest and no competing financial interests.
