# Supplementary material for: Focal-adhesion kinase regulates the sialylation of N-glycans via the PI4KIIα-PI4P pathway
Source: J Biol Chem. 2023 Jul 13;299(8):105051. doi: 10.1016/j.jbc.2023.105051 (PMC10406863; doi:10.1016/j.jbc.2023.105051)
Supplement: Supporting Figures S1–S4 [file mmc1.pdf]

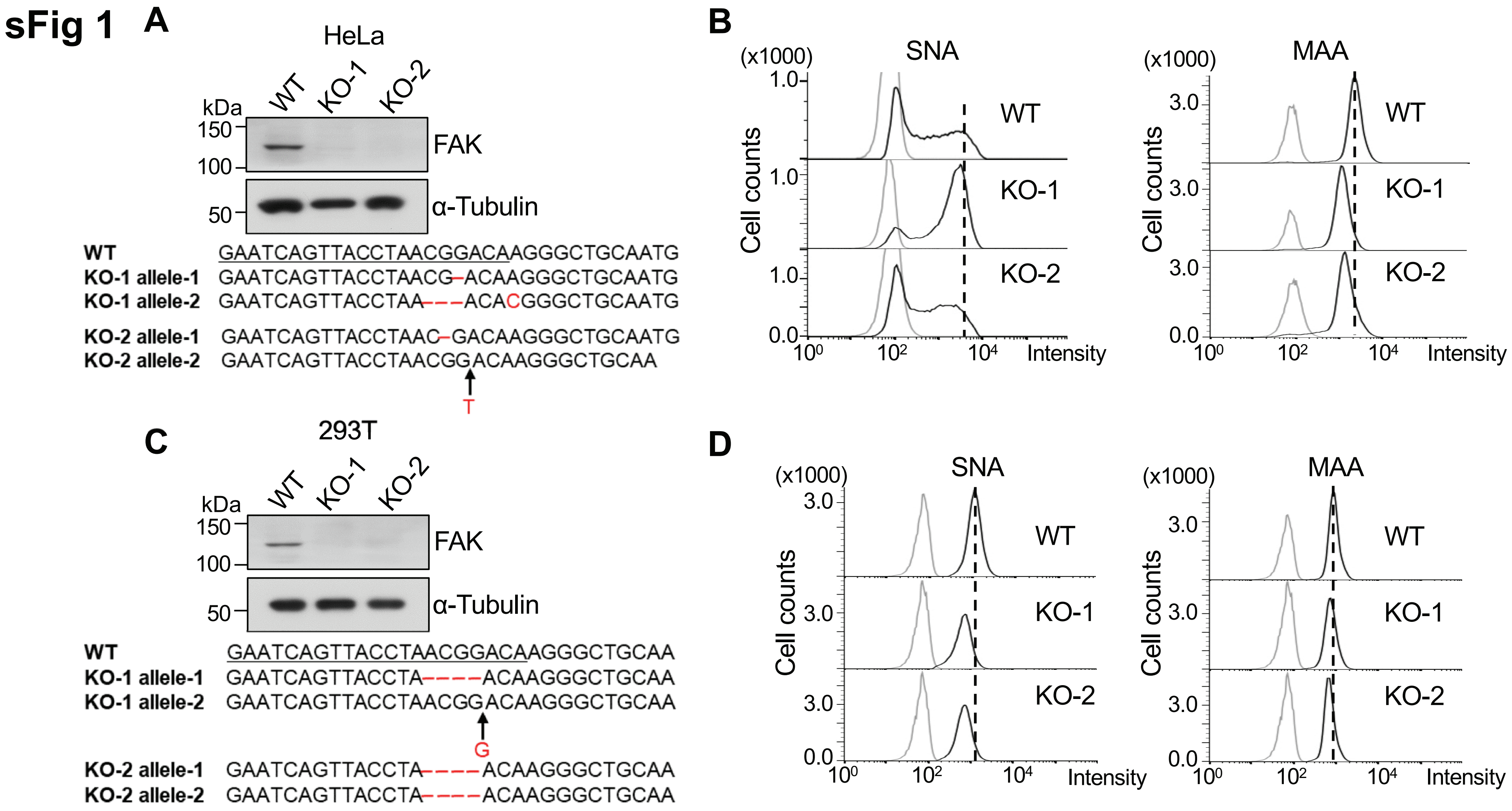

sFig.1. Alteration of sialylation in FAK KO HeLa cells and 293T cells

A, Cell lysates were extracted from the WT and two FAK KO cell clones (KO-1and KO-2) in HeLa cells. Equal amounts of cell lysates were loaded into 7.5% SDS-PAGE gel to detect the expression of FAK (upper layer). The FAK-targeting gRNA was designed (underlined). Compared to WT cells, the sequence of FAK KO-1 HeLa cells showed one base (G) deletion in allele 1 and 3-base (CGG) deletion and a mutation (A was replaced by C in the red letter) in allele 2; the sequence of FAK KO-2 HeLa cells showed one base (G) deletion in allele 1 and an insertion mutation (T inserted between G and A) in allele 2. B, Comparison of the sialylation levels on the cell surface among the WT, FAK KO-1, and FAK KO-2 HeLa cells by flow cytometry analysis. The same numbers of cells were incubated with SNA and MAA. C, Expression levels of FAK in 293T cells were detected by western blot. Equal proteins were subjected to 7.5% SDS-PAGE gel (upper layer). The FAK sequence exhibited 4-base (ACGG) deletion in allele 1 and an insertion mutation (G inserted between G and A) in allele 2 in the KO-1 293T cells, and 4-base (ACGG) deletion in the KO-2 293T cells compared to the WT cells. D, Comparison of the sialylation levels on the cell surface among the WT, FAK KO-1, and FAK KO-2 293T cells were also detected by flow cytometry analysis using SNA and MAA lectins described as "Experimental procedures".

sFig 2

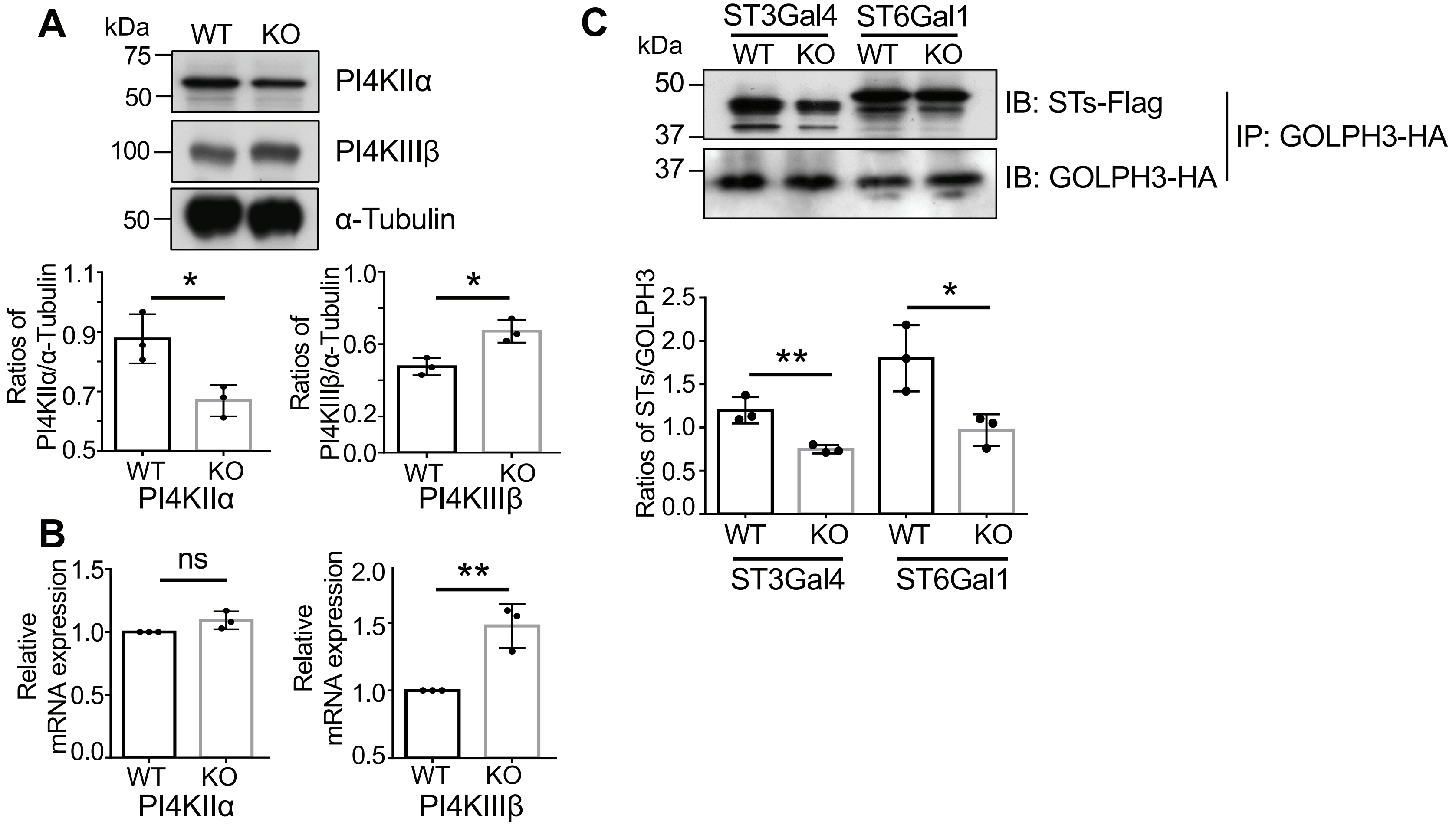

sFig.2. Effects of FAK on PI4Ks and complex formation between GOLPH3 and sialyltransferases in FAK KO 293T cells

A, Expression levels of PI4KIIα and PI4KIIIβ in 293T cells were detected by western blot. Equal proteins were subjected to 7.5% SDS-PAGE gel and verified by indicated antibodies. α-Tubulin was used as a loading control. Data were quantified by Image J software and obtained from three independent experiments. All values represent using unpaired Student *t* test analysis as the mean ± SD. *$p < 0.05$. B, The mRNA levels of PI4KIIα and PI4KIIIβ in 293T cells were detected by qPCR. GAPDH was used as an internal control. All values were normalized to that of the GAPDH. Data were represented as the mean ± SD from three independent experiments. The ratio of PI4KIIα/GAPDH or PI4KIIIβ/GAPDH in WT cells was set as 1.0. ns, no significance, $p > 0.05$; **$p < 0.01$. C, Effects of FAK on complex formation between GOLPH3 and STs. Equal amounts of proteins of 293T cells (WT, FAK KO) co-transfected with GOLPH3-HA and ST3Gal4-Flag or ST6Gal1-Flag, were immunoprecipitated with anti-HA-agarose, and the immunoprecipitates were detected by the anti-Flag antibody. The experiments were independently repeated three times. The relative intensities were calculated by the intensities of total sialyltransferases (ST3Gal4 or ST6Gal1)-Flag against GOLPH3-HA. All values were analyzed by unpaired Student *t* test analysis and presented as the mean ± SD. *$p < 0.05$; **$p < 0.01$.

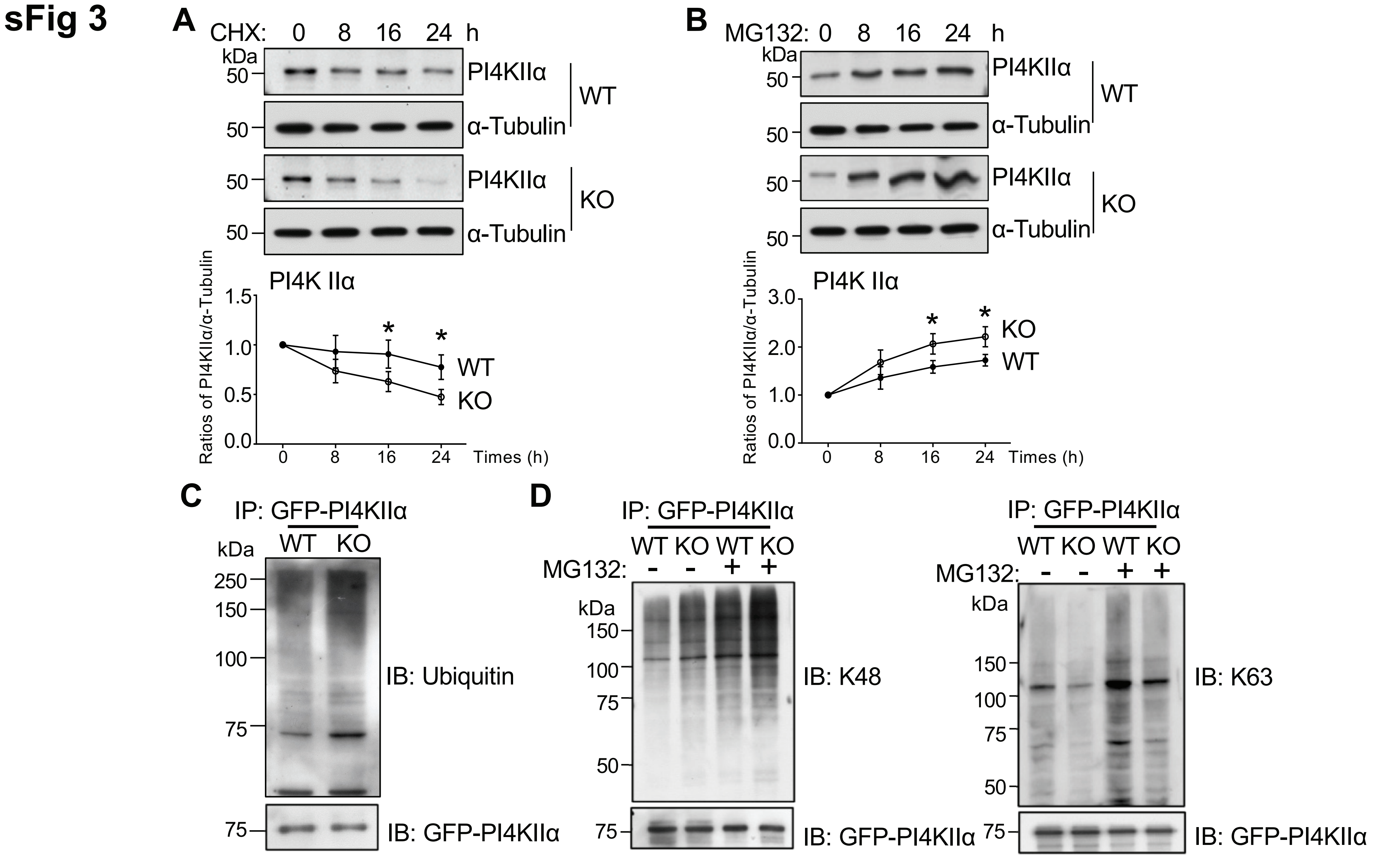

sFig.3. Effects of FAK on PI4KIIα stability in 293T cells

The WT and FAK KO 293T cells were treated with 50 mM CHX (A), or 30 μM MG132 (B), for indicated times. Equal cell lysates were used for western blotting with an anti-PI4KIIα antibody. α-Tubulin was used as a loading control. The relative values were calculated by the density of PI4KIIα at each indicated time point against the density of that at time 0 h (without treatment). The ratio at the 0 h point was set as 1.0. Values were presented as the mean ± SD from three independent experiments. $*p < 0.05$. The WT and FAK KO 293T cells were transfected with GFP-PI4KIIα for 48 h and treated with (+) or without (-) MG132, a proteasome inhibitor. Equal amounts of proteins were used to immunoprecipitate with anti-GFP antibody beads, and the immunoprecipitates were western blotted with anti-ubiquitin antibody (C), and with anti-K48 or anti-K63 ubiquitin antibody (D). GFP-PI4KIIα was used as a loading control.

sFig 4

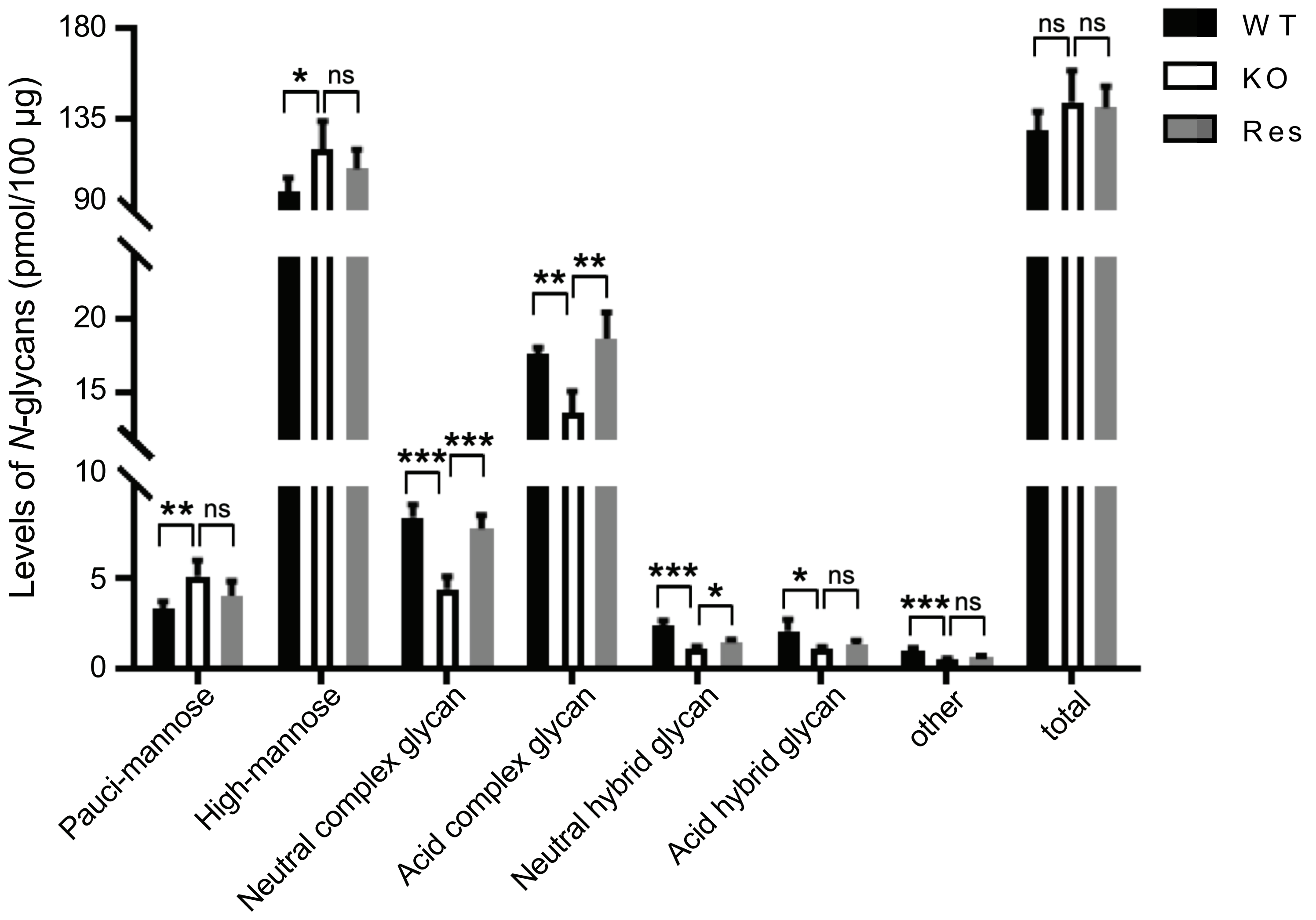

sFig.4. Statistical analyses of *N*-glycans in HeLa cells analyzed by MALDI-TOF MS spectra

The expression levels of *N*-glycans in HeLa cells analyzed by MALDI-TOF MS spectra were showed in Table 1. The statistical analyses of the amount of *N*-glycans per 100 µg protein were represented as the mean ± SD from four independent experiments. All values were analyzed by one-way ANOVA with Tukey's *post hoc* test. ns, no significance, $p > 0.05$; $*p < 0.05$; $**p < 0.01$; $***p < 0.001$.
